# Risk factors of aggressive behavior in female patients with schizophrenia

**DOI:** 10.3389/fpsyt.2026.1839475

**Published:** 2026-08-26

**Authors:** Furong Tang, Zhenzhong Shen, Yongxing Zhu, Jiajia Wang, Lin Zhang

**Affiliations:** 1 Department of Psychiatry, The Affiliated Xuzhou Oriental Hospital of Xuzhou Medical University, Xuzhou, Jiangsu, China; 2 School of Nursing, Xuzhou Medical University, Xuzhou, Jiangsu, China

**Keywords:** aggressive behavior, female, risk factor, schizophrenia, violence

## Abstract

**Objective:**

To investigate the risk factors associated with violent behavior in female patients with schizophrenia.

**Methods:**

A total of 85 female patients with schizophrenia were assessed using the Modified Over Aggression Scale (MOAS), the Positive and Negative Symptoms Scale (PANSS), the Childhood Trauma Questionnaire (CTQ), and the Psychopathy Checklist‐Revised (PCL‐R). Based on their MOAS scores, patients were divided into non-violent and violent groups. Univariate analysis and binary logistic regression were conducted to identify the risk factors for aggressive behavior.

**Results:**

Violent patients exhibited significantly higher scores than non-violent patients in terms of positive symptom, paranoid syndrome and aggressive syndrome of PANSS, emotional abuse and sexual abuse of CTQ, and antisocial features of PCL‐R. Moreover, logistic analysis revealed that positive symptom (OR = 1.506, 95% CI 1.164-1.947, *P* = 0.002), paranoia (OR = 1.776, 95% CI 1.114-2.833, *P* = 0.016), aggressive syndrome (OR = 1.734, 95% CI 1.047-2.872, *P* = 0.033), antisocial tendency (OR = 1.679, 95% CI 1.012-2.785, *P* = 0.045) and emotional abuse (OR = 1.783, 95% CI 1.187-2.678, *P* = 0.005) were the risk factors for the occurrence of aggression in female patients with schizophrenia.

**Conclusion:**

This study highlighted the significant association between violence in female schizophrenia patients and psychopathy traits, childhood trauma as well as personality characteristics. These findings underscored the potential utility of incorporating PCL-R and CTQ assessments into the clinical evaluation of aggression in this population.

## 1 Introduction

Schizophrenia is a severe mental disorder affecting approximately 1% of people worldwide (1), and is characterized by significant impairments in cognition, emotion, thought, and behavior. Some patients exhibit aggressive behavior, presenting as agitation, impaired impulse control, and diminished social sensitivity. A follow-up investigation of 1,435 schizophrenia patients reported that 5.4% had committed violent conduct (2). Although infrequent, individuals with schizophrenia have a higher risk of violence than the general population (3, 4). It was reported that the risk of aggression in patients with schizophrenia was 10.7-fold higher than that of the general population (5). Moreover, male patients with schizophrenia had a 4-fold higher risk of violent crime compared with the general population, whereas female patients had an 8-fold higher risk (4). Another study similarly demonstrated gender differences in violent behavior among patients with schizophrenia, with women showing higher frequency of aggression and men more often committing severe violent conduct (6). Their aggressive behavior will cause a multitude of negative consequences for victims and the perpetrator. Understanding the risk factors for violence may help the evidence-based identification and prediction of aggressive conduct.

The aggressive behavior of people with schizophrenia has long been a public concern. Studies have shown that socio-demographic factors, including age at onset, marital status and illness history, are related to the occurrence of violent behavior in individuals with schizophrenia. Patients with an earlier age at onset, who are unmarried, and lower levels of education have an increased risk of committing aggression (7, 8). Clinical symptoms also show a strong association with violent behavior in patients with schizophrenia. Although the relationship between negative symptoms and aggression remains unclear, a growing body of research has revealed that positive symptoms are associated with a higher risk of aggression (9). In addition, childhood trauma, an environmental factor, has been reported to contribute to violent behavior in schizophrenia (10). Furthermore, personality traits are also associated with aggressive behavior in men with schizophrenia (11). A comprehensive assessment of risk factors for aggression is helpful for its prevention and early intervention.

Most prior studies on violence in schizophrenia have focused on male patients. Given gender differences in personality traits and crime rates, the risk factors for aggression may differ between men and women. Recent work on involuntarily committed female offenders with mental disorders provides a valuable methodological precedent (12), yet population-specific risk factors in female inpatients with schizophrenia are still underexplored. To date, however, the risk factors for violent conduct in female patients with schizophrenia remain underexplored. The present study therefore aimed to systematically examine the features and associated factors of aggression in female patients with schizophrenia, thereby providing a theoretical basis for the early prevention, identification, and intervention of such behavior.

## 2 Methods

### 2.1 Participants

This study was conducted at Xuzhou Oriental People’s Hospital from November 2024 to August 2025. Inclusion criteria: (1) female inpatients aged 18–60 years; (2) met the diagnostic criteria for schizophrenia in the International Statistical Classification of Diseases and Related Health Problems 10th Revision (ICD-10); (3) provided written informed consent from the patient or their legal guardians. Exclusion criteria: (1) with mental disorders or intellectual disabilities due to organic brain or physical diseases; (2) with a history of brain trauma or neurological disorders; (3) unable to understand or complete the questionnaire.

All participants were divided into violent and non-violent groups according to the score of Modified Over Aggression Scale (MOAS). A self-designed questionnaire was used to collect data on participants’ age, illness duration, marital status, education level, and family history. The study protocol was approved by the Ethics Committee of Xuzhou Oriental People’s Hospital (XZSDFRMYY2022-BG-07).

### 2.2 Instruments

#### 2.2.1 Modified over aggression scale

Aggressive behaviors were assessed using the MOAS, which comprises four subscales: verbal aggression, physical aggression, self-aggression, and aggression toward others. Each subscale is scored from 0 to 4, with higher scores indicating greater severity. The total MOAS score, ranging from 0 to 40, was then calculated as the weighted sum for the four subscales, as described previously (8). In the present study, classification into violent and non-violent groups was based on established MOAS cutoff criteria in aggression research (13–16). Specifically, patients with a total score of ≥ 5 or an object aggression score of > 1 were assigned to the violent group, while all others were assigned to the non-violent group.

#### 2.2.2 Positive and negative syndrome scale

The PANSS was administered to evaluate symptom severity on the day of admission. The scale yields scores for positive (7 items), negative (7 items), and general psychopathology (16 items) subscales (7, 17). Each item is rated on a 7-point Likert scale (1-7), with higher scores indicating greater symptom severity. In this study, these items were aggregated into six symptom domains following the clinical classification outlined in the Chinese Handbook of Psychiatric Rating Scales (18): response deficit, thought disorder, activation, paranoia, depression, and aggression (19, 20).

#### 2.2.3 Childhood trauma questionnaire

The CTQ is a self-report instrument designed to retrospectively assess participants’ experiences of childhood trauma. The scale consists of 28 items, including 25 assessment items and 3 validity items, and assesses five subtypes of trauma: emotional abuse, physical abuse, sexual abuse, emotional neglect, and physical neglect. Each item is scored from 1 (none) to 5 (always), with higher scores indicating more severe maltreatment. Established cutoff scores are available to determine the presence or absence of trauma (21).

#### 2.2.4 Psychopathy checklist-revised

The PCL-R is a widely used instrument for evaluating psychopathy, and contains 20 items rated on a 3-point Likert scale (0-2), with total scores ranging from 0 to 40. Except for item 11 and 17, these items are organized into four factors including interpersonal factors, affective factors, lifestyle factors, and antisocial factors. A higher total score is associated with a greater likelihood of violence.

### 2.3 Statistical analysis

All statistical analyses were performed using SPSS software (version 25.0; IBM, Inc., Chicago, Illinois). Categorical variables are expressed as frequencies (n) and percentages (%), and compared using the Chi-squared test. Continuous variables are represented as mean ± standard deviation (SD), and analyzed by independent sample t-test. Variables demonstrating statistical significance in the univariate analysis subsequently included in the stepwise logistic regression analysis to identify independent risk factors for aggressive behavior. A two-tailed *P* < 0.05 was considered statistically significant.

## 3 Results

### 3.1 Demographic characteristics of the survey subjects

A total of 85 female patients who met the study eligibility were enrolled, comprising 43 violent and 42 non-violent patients. As shown in **Table 1**, there was no significant difference between the two groups in demographic data, including age, illness duration, marital status, education level, family history and mean daily antipsychotic dosage (expressed as chlorpromazine equivalents) (*P* > 0.05).

**Table 1:** Comparison of socio-demographics between the violent and non-violent groups.

Variable	Non-violent group (n = 42)	Violent group (n = 43)	Statistics	*P*
Age (year)	42.45 ± 9.80	40.02 ± 10.50	t = 1.102	0.274
Duration of illness (year)	6.88 ± 5.70	8.09 ± 7.56	t = -0.833	0.407
Marital status				
Single	8 (19.0%)	9 (20.9%)		
Married	23 (54.8%)	18 (41.9%)	χ² = 1.583	0.453
Divorced	11 (26.2%)	16 (41.9%)		
Level of education				
Primary school or below	7 (16.7%)	4 (9.3%)		
Secondary school	5 (11.9%)	15 (34.9%)	χ² = 6.595	0.083
High school/specialized secondary school	27 (64.3%)	21 (48.8%)		
College and above	3 (7.1%)	3 (7.0%)		
Family history				
Yes	1 (2.38%)	2 (4.65%)	χ² = 0.322	1.000
No	41 (97.62%)	41 (95.35%)		
Antipsychotic dose* (mg/day)	392.64 ± 155.70	449.86 ± 137.66	t = 1.793	0.076

*Doses of antipsychotics: the received dose of antipsychotics expressed in chlorpromazine equivalents.

### 3.2 Comparison of the PANSS, CTQ and PCL-R scores

Intergroup comparison of the PANSS showed that the violent group had significantly higher scores on positive symptoms (28.44 vs. 26.36, *P* = 0.001), paranoia (12.98 vs. 12.00, *P* = 0.004), and aggressiveness (16.44 vs. 15.79, *P* = 0.027), compared with the non-violent group. No other factors differed significantly between the two groups (**Table 2**).

**Table 2:** Comparison of the PANSS scores between the violent and non-violent groups.

Variable	Non-violent group (n = 42)	Violent group (n = 43)	Statistics	*P*
Total score	93.38 ± 8.37	95.51 ± 8.65	-1.154	0.252
Positive symptom scale	26.36 ± 2.32	28.44 ± 3.25	-3.402	**0.001**
Negative symptom scale	19.64 ± 4.29	18.77 ± 6.20	0.759	0.452
General psychopathology scale	47.38 ± 5.17	48.3 ± 4.72	-0.858	0.393
Response deficit syndrome	8.52 ± 1.97	9.02 ± 3.38	-0.836	0.406
Thought disorder syndrome	14.79 ± 2.33	15.77 ± 2.49	-1.876	0.064
Activation syndrome	9.71 ± 1.40	9.95 ± 1.40	-0.788	0.433
Paranoid syndrome	12.00 ± 1.43	12.98 ± 1.63	-2.942	**0.004**
Depressive syndrome	10.29 ± 3.45	10.33 ± 2.44	-0.062	0.951
Aggressive syndrome	15.79 ± 1.41	16.44 ± 1.28	-2.252	**0.027**

*P*-values < 0.05 were marked in bold.

The total CTQ score and its subscales scores are presented in **Table 3**. Compared with the non-violent group, higher scores were observed in the violent group regarding CTQ total score (39.8 vs. 36.55, *P* = 0.024), emotional abuse (7.37 vs. 6.17, *P* = 0.016), and sexual abuse (6.67 vs. 5.52, *P* = 0.023). There was no significant differences between the two groups in the other subtypes.

**Table 3:** Comparison of the CTQ scores between the violent and non-violent groups.

Variable	Non-violent group (n = 42)	Violent group (n = 43)	Statistics	*P*
Total score	36.55 ± 5.99	39.81 ± 7.01	-2.306	**0.024**
Emotional abuse	6.17 ± 1.82	7.37 ± 2.62	-2.469	**0.016**
Physical abuse	5.95 ± 1.38	6.00 ± 1.22	-0.169	0.866
Sexual abuse	5.52 ± 1.42	6.67 ± 2.91	-2.326	**0.023**
Emotional neglect	9.86 ± 3.24	10.26 ± 2.73	-0.614	0.541
Physical neglect	9.05 ± 2.57	9.51 ± 2.78	-0.799	0.427

*P*-values < 0.05 were marked in bold.

As shown in **Table 4**, the total score (9.35 vs. 7.24, *P* = 0.018) and antisocial factor (2.42 vs. 1.52, *P* = 0.003) in the PCL-R were significantly higher in the violent group than the non-violent group. No significant differences were observed between the two groups in the other factors.

**Table 4:** Comparison of the PCL-R scores between the violent and non-violent groups.

Variable	Non-violent group (n = 42)	Violent group (n = 43)	Statistics	*P*
Total score	7.24 ± 3.85	9.35 ± 4.19	-2.417	**0.018**
Interpersonal factor	0.71 ± 1.19	0.88 ± 1.22	-0.647	0.520
Affective factor	1.55 ± 1.58	2.00 ± 2.13	-1.111	0.270
Lifestyle factor	3.45 ± 2.00	4.05 ± 1.94	-1.390	0.168
Antisocial factor	1.52 ± 1.40	2.42 ± 1.33	-3.019	**0.003**

*P*-values < 0.05 were marked in bold.

### 3.3 Ordinal logistic regression analysis of risk factors for aggression

To identify independent risk factors of aggressive behaviors, a stepwise ordinal logistic regression analysis was conducted on variables that showed significant intergroup difference in univariate analysis (**Table 5**). The results demonstrated that higher scores on the following factors were associated with an increased risk of aggression in female patients with schizophrenia: positive symptom (OR = 1.506, 95% CI 1.164-1.947, *P* = 0.002), paranoia (OR = 1.776, 95% CI 1.114-2.833, *P* = 0.016), aggressive syndrome (OR = 1.734, 95% CI 1.047-2.872, *P* = 0.033), antisocial tendency (OR = 1.679, 95% CI 1.012-2.785, *P* = 0.045) and emotional abuse (OR = 1.783, 95% CI 1.187-2.678, *P* = 0.005).

**Table 5:** Logistic regression analysis.

Factor	OR	95% CI	*P*
Positive symptom scale	1.506	1.164-1.947	**0.002**
Paranoid syndrome	1.776	1.114-2.833	**0.016**
Aggressive syndrome	1.734	1.047-2.872	**0.033**
Emotional abuse	1.783	1.187-2.678	**0.005**
Sexual abuse	1.352	0.937-1.950	0.107
Antisocial factor	1.679	1.012-2.785	**0.045**

P-values < 0 .05 were marked in bold.

## 4 Discussion

Although aggressive behavior is uncommon in people with schizophrenia, this population has a higher risk of aggression than the general population (22). Previous studies have described the characteristics and related factors of aggressive behaviors in male individuals with schizophrenia (11, 19), yet the factors influencing aggressiveness in female schizophrenia patients are still not well understood. This study focused on female patients with schizophrenia and investigated the risk factors for violent behaviors. We found that patients in the aggressive group had higher scores on positive symptom, paranoid syndrome and aggressive syndrome of PANSS, emotional abuse and sexual abuse of CTQ, and antisocial features of PCL‐R. Moreover, our findings demonstrated that positive symptom, paranoia, aggressive syndrome, antisocial tendency and emotional abuse were risk factors for aggression. The present study highlighted the necessity of using the PANSS, PCL-R and CTQ in the assessment of female patients with schizophrenia. It is noteworthy that these findings should be interpreted as indicators of relative risk rather than absolute symptom exacerbation.

In the present study, no significant differences were observed between violent and non-violent patients in terms of socio-demographic variables, including age, duration of illness, marital status, education level, and family history of psychiatric disorders. While previous research has showed that lower education levels (8), being unmarried (7) and higher doses of antipsychotics (23) are associated with an increased risk of aggression, others have reported no such correlations (13, 19). A plausible explanation for these discrepant findings is that female schizophrenia patients are more likely to show exhibit less severe violence (6), which may have little impact on their educational attainment or marital status. Nevertheless, our null findings should be interpreted with caution, as the modest sample size may have limited statistical power to detect subtle group differences.

Clinical symptoms, as measured by the PANSS, show a stronger association with violent behaviors than demographic factors (24). In line with previous studies (20, 25), our data indicated that positive symptoms, especially persecutory delusions, emerged as key drivers of aggression in our cohort. Similarly, it has been reported that the emergence of persecutory delusions explains violent behaviors in untreated patients with schizophrenia (26). Rather than goal-directed violence, such aggression is better conceptualized as a maladaptive defensive response, whereby individuals attempt to neutralize a perceived threat. This pattern is consistent with cognitive models suggesting that paranoid individuals tend to overattribute hostile intent to ambiguous social cues (27). Given the high prevalence of persecutory delusions in schizophrenia patients (28), our findings underscore the importance of routinely assessing delusion-driven threat appraisal-particularly in female patients, for whom such appraisals may represent a more proximal marker of imminent risk than static demographic variables.

Although the etiology of schizophrenia remains unclear, accumulating evidence has suggested that it is a neurodevelopmental disorder resulting from the interplay of genetic, psychosocial, and environmental factors (29, 30). Childhood trauma, also known as childhood adversity, has been identified as an important environmental contributor to the pathophysiology of schizophrenia (21, 31). In line with prior research (32), emotional abuse emerged as a distinct risk factor for aggression in our cohort, whereas emotional neglect did not differ between groups. Consistently, an early research revealed that emotional abuse is more strongly correlated with various mental disorders than emotional neglect, another form of maltreatment (33). This disparity may reflect differences in cognitive attribution: whereas emotional abuse directly imposes negative self-cognitions (e.g., “you are worthless”), neglect requires the child to generate endogenous attributions for deprivation (34). Childhood abuse results in an unmet need for security and self-worth, and hinders the integration of self and object images. This, in turn, leads to difficulties in accurately processing social information during interpersonal interactions, and fosters a hostile attribution bias, thereby increasing the likelihood of aggressive behaviors. These findings underscore the clinical utility of the CTQ, particularly the emotional abuse subscale, in violence risk assessments for female patients with schizophrenia.

Antisocial personality traits represent a well-established risk factor for aggression (35, 36). In the present study, the antisocial factor of the PCL-R independently predicted violent behavior among female patients with schizophrenia, extending prior evidence from male cohorts (11). Aggression in schizophrenia may follow at least two distinct pathways: one linked to premorbid conditions (e.g., antisocial behavior), and the other to the acute psychopathology of the illness (37). People with preexisting conduct disorders may develop antisocial attitudes and thinking patterns, and lack appropriate social skills early in life (38). Their aggression pattern may differ from that of individuals whose aggressive behavior emerged following the onset of schizophrenia (39). Given that comorbid antisocial personality disorder further amplifies the propensity for violent crime (40), our results underscore that personality disorders, particularly as quantified by the PCL-R, should be taken into account in the assessment and management of violence in schizophrenia.

The present study has several limitations that should be considered. First, the cross-sectional design precludes causal inferences, restricting our findings to associations. Consequently, it remains unclear whether identified risk factors temporally preceded or resulted from aggressive behavior; this limitation underscores the need for longitudinal investigations to establish temporal precedence. Second, the reliance on retrospective self-report instruments, particularly the CTQ, introduces recall bias, which may be especially pertinent in schizophrenia due to cognitive and metacognitive impairments. potentially leading to either the exaggeration or minimization of reported traumatic experiences. Third, the relatively small sample size limits the generalizability of the findings, and could explain the absence of demographic differences, underscoring the need for larger, multi-center studies. Fourth, this study lacked biological and neurocognitive data, which constitutes a notable limitation. While our findings highlight the roles of trauma and personality traits, the absence of objective biomarkers and neuropsychological profiles prevents a deeper understanding of the underlying neurobiological mechanisms. Finally, as this study focused exclusively on female inpatients in a specific cultural context, the results may not be directly applicable to male patients or populations in different sociocultural environments.

## 5 Conclusion

This study employed multiple assessment tools, including the PANSS, PCL-R and CTQ, to investigate risk factors for violent behavior in female patients with schizophrenia. Our findings indicate that the factors influencing aggression in this population are complex and multifactorial. Specifically, positive symptom, paranoia and aggressive syndrome in PANSS, antisocial factor in PCL-R, as well as emotional abuse in CTQ may serve as predictors of aggression. A comprehensive assessment that integrates clinical symptoms, childhood trauma and personality characteristics could be valuable for identifying female schizophrenia patients at elevated risk of aggressive behavior.

## Data Availability

The original contributions presented in the study are included in the article/supplementary material. Further inquiries can be directed to the corresponding author.
